# Long-Term Effect of β-Blocker Use on Clinical Outcomes in Postmyocardial Infarction Patients: A Systematic Review and Meta-Analysis

**DOI:** 10.3389/fcvm.2022.779462

**Published:** 2022-04-08

**Authors:** Chunling Liang, Chenhao Zhang, Shibao Gan, Xiaojie Chen, Zhihui Tan

**Affiliations:** ^1^Department of Emergency, Wangjing Hospital of China Academy of Chinese Medical Sciences, Beijing, China; ^2^Department of General Practice, 920th Hospital of Joint Logistics Support Force of Chinese People's Liberation Army, Kunming, China

**Keywords:** β-blocker, clinical outcomes, meta-analysis, post-myocardial infarction, systematic review

## Abstract

**Background:**

Prior studies provided inconsistent results regarding long-term effect of β-blocker use on clinical outcomes in postmyocardial infarction (MI) patients.

**Methods:**

We searched for articles regarding long-term effect of β-blocker use on clinical outcomes in patients after MI and published them before July 2021 in the databases as follows: PubMed, Web of Science, MEDLINE, EMBASE, and Google Scholar. STATA 12.0 software was used to compute hazard ratios (HRs) and their 95% confidence intervals (CIs).

**Results:**

The study indicated that β-blocker group had significantly lower long-term all-cause mortality, cardiovascular mortality, major adverse cardiac events (MACEs) in post-MI patients, compared to no β-blocker group (all-cause mortality: HR, 0.67; 95% CI: 0.56–0.80; cardiovascular mortality: HR, 0.62; 95% CI: 0.49–0.78; MACE: HR, 0.87; 95% CI: 0.75–1.00). The study indicated no significant long-term effect of β-blocker use on risk of hospitalization for heart failure (HF), risk of recurrent MI, risk of stroke, and risk of repeat revascularization in post-MI patients (risk of hospitalization for HF: HR, 0.82; 95% CI: 0.58–1.16; risk of recurrent MI: HR, 0.93; 95% CI: 0.78–1.11; risk of stroke: HR, 0.94; 95% CI: 0.79–1.12; risk of repeat revascularization: HR, 0.91; 95% CI: 0.80–1.04).

**Conclusions:**

The meta-analysis demonstrated significant long-term effects of β-blocker use on all-cause mortality, cardiovascular mortality, and risk of MACE in post-MI patients, whereas no significant long-term effect was shown on risk of hospitalization for HF, risk of recurrent MI, risk of stroke, and risk of repeat revascularization in post-MI patients.

## Introduction

Myocardial infarction (MI) is one of the most common causes of mortality in the world and results in over a third of deaths in developed nations annually (1–6). In spite of the effective therapy strategies, the overall survival for MI patients has maintained almost unchanged with the increasing number of MI patients (7). Effective therapy for post-MI patients is essential to prevent recurrence of MI, cardiac death, stroke, and other major adverse cardiac events (MACEs).

In recent decades, β-blocker use has become a key part of secondary prevention following MI (8), especially in high-risk patients, such as those with low left ventricular ejection fraction (LVEF) (9). But currently, the role of β-blocker use in the treatment of MI could be raised question. Recently, some studies (10, 11) showed a long-term benefit of β-blocker use on all-cause mortality in post-MI patients, whereas some studies (12, 13) showed that β-blocker use had no long-term benefit on all-cause mortality in post-MI patients. Considering β-blocker is a clinical common drug, it is necessary to make clear the role of β-blocker use on clinical outcomes in post-MI patients. A recent meta-analysis (14) showed that there is no association between β-blocker use and all-cause mortality in post-MI patients. However, the meta-analysis showed a significant publication bias (Egger's test: *p* = 0.001). To provide more evidence to confirm the effect of β-blocker use on all-cause mortality in post-MI patients, this study aimed to make an updated meta-analysis for the previous meta-analysis regarding the long-term benefit of β-blocker use on all-cause mortality in post-MI patients. In addition, the study aimed to explore the long-term effect of β-blocker use on other clinical outcomes (including cardiovascular mortality, risk of hospitalization for heart failure (HF), risk of recurrent MI, risk of MACE, risk of stroke, and risk of repeat revascularization) in post-MI patients.

## Methods

The study was performed based on the Preferred Reporting Items for Systematic reviews and Meta-Analysis (PRISMA) guideline (15).

### Search Strategy

We searched for articles regarding long-term effect of β-blocker use on clinical outcomes in patients after MI and published them before July 2021 in the databases as follows: PubMed, Web of Science, MEDLINE, EMBASE, and Google Scholar. We used the following search terms: (“myocardial infarction” OR “MI”) AND (“β-blocker” OR “β blocker” OR “β1-blocker” OR “beta-blocker” OR “beta-blocker” OR “beta-adrenoceptor blockade” OR “beta-adrenergic blockade” OR “beta blockade” OR “betablocker”).

### Inclusion Criteria and Exclusion Criteria

Inclusion criteria were as follows: (1) we included randomized controlled trials or observational studies exploring the long-term effect of β-blocker use on clinical outcomes in patients after MI; (2) median follow-up duration was equal to or more than 6 months. Additionally, studies were excluded based on the following exclusion criteria: (1) we excluded articles that did not provide sufficient data for hazard ratios (HRs) and their 95% confidence intervals (CIs) regarding the long-term effect of β-blocker use on all-cause mortality, cardiovascular mortality, risk of hospitalization for HF, risk of recurrent MI, risk of MACE, risk of stroke, or risk of repeat revascularization in patients after MI. (2) meta-analyses, reviews, and case reports. All the abstracts and full texts were read independently by two researchers (Chunling Liang and Chenhao Zhang). When the inconsistencies in the study selection appeared, the articles were discussed and decided by the three authors (Chunling Liang, Chenhao Zhang, and Shibao Gan). Additionally, regarding the long-term effect of β-blocker use on clinical outcomes in patients after MI with low EF, the study included studies where none or only a minority of patients had LVEF < 40% at baseline.

### Data Collection

We collected data from included studies. These data included the followings: author name, publication year, study type, study location, sample size, mean age, gender, ratio of ST elevation myocardial infarction (STEMI), ratio of patients treated with percutaneous coronary intervention (PCI), LVEF, ratio of history of HF, ratio of Killip class ≤2, ratio of history of hypertension, ratio of history of diabetes, ratio of history of smoking, ratio of prior MI, ratio of treatment with angiotensin receptor blockers (ARBs)–angiotensin-converting enzyme inhibitors (ACEI), ratio of treatment with acetylsalicylic acid (ASA), ratio of treatment with statins, and follow-up duration. Hospitalization for HF was defined as hospitalization because of worsening HF requiring intravenous drug therapy. Recurrent MI was defined as recurrent symptoms and new electrocardiograph (ECG) changes that were compatible with MI or cardiac markers that were expressed at least two times the upper limit of normal.

### Statistical Analysis

STATA 12.0 software was used to compute HRs and 95% CIs regarding the long-term effect of β-blocker use on clinical outcomes in patients after MI. Q test and I^2^ were applied to evaluate heterogeneities between included studies. With high heterogeneity (*p* ≤ 0.05 and I^2^ ≥ 50%) between included studies, random-effects models were used as computation methods; on the contrary, with invariably low heterogeneity (*p*-value for Q test > 0.05 and I^2^ < 50%) between included studies, fixed effects models were used as computation methods. In addition, subgroup analyses (for different research designs and ethnicities) were used to explore the source of the heterogeneity between included studies. Meta-regression analyses for variables were conducted to explore source of the heterogeneity. These variables included the followings: publication year, age, gender, ratio of STEMI, ratio of patients treated with PCI, LVEF, ratio of history of HF, ratio of Killip class ≤2, ratio of history of hypertension, ratio of history of diabetes, ratio of history of smoking, ratio of prior MI, ratio of treatment with ARBs/ACEI, ratio of treatment with ASA, ratio of treatment with statins, and follow-up duration. Sensitivity analyses were used to assess the stabilization of meta-analysis. Moreover, Begg's test, Egger's test, and funnel plot were used to assess publication bias. We conducted quality assessments of the included studies to systematically assess their most important biases and weaknesses. We used the Newcastle-Ottawa scale (16) to evaluate the quality of the observational studies.

## Results

### Characteristics of Included Studies

Supplementary Table S1 shows study characteristics of 29 finally included studies (9–13, 17–40). Supplementary Figure S1 illustrated the selection process. This study included 22 retrospective studies (9, 10, 13, 17–20, 22, 24–30, 33–38, 40) (including 1,73,438 β-blocker users and 31,836 no β-blocker users) and 7 prospective studies (11, 12, 21, 23, 31, 32, 39) (including 22,557 β-blocker users and 14,182 no β-blocker users). Among the included studies, 24 studies (9–13, 19, 21–25, 27–38, 40) (including 1,81,757 β-blocker users and 37,695 no β-blocker users) were included to explore the long-term effect of β-blocker use on all-cause mortality in patients after MI. A number of 11 studies (12, 17, 20, 24, 26, 27, 29, 30, 36, 39, 40) (including 23,172 β-blocker users and 12,220 no β-blocker users) were included to explore the long-term effect of β-blocker use on cardiovascular mortality in patients after MI. A number of five studies (10, 11, 13, 20, 27) (including 13,900 β-blocker users and 14,525 no β-blocker users) were included to explore the long-term effect of β-blocker use on risk of hospitalization for HF in patients after MI. A number of nine studies (13, 20, 26–30, 39, 40) (including 26,917 β-blocker users and 13,869 no β-blocker users) were included to explore the long-term effect of β-blocker use on risk of recurrent MI in patients after MI. A number of 10 studies (18, 19, 25–28, 30, 33, 36, 40) (including 12,374 β-blocker users and 10,302 no β-blocker users) were included to explore the long-term effect of β-blocker use on risk of MACE in patients after MI. A number of three studies (13, 26, 29) (including 10,783 β-blocker users and 5,885 no β-blocker users) were included to explore the long-term effect of β-blocker use on risk of stroke in patients after MI. A number of three studies (29, 30, 39) (including 14,968 β-blocker users and 3,935 no β-blocker users) were included to explore the long-term effect of β-blocker use on risk of repeat revascularization in patients after MI.

Regarding the long-term effect of β-blocker use on clinical outcomes in patients after MI with low EF, this study included 16 studies (9, 12, 19, 23, 25, 27–30, 33–38, 40) for all-cause mortality, eight studies (12, 26, 27, 29, 30, 36, 39, 40) for all-cause mortality, seven studies (26–30, 39, 40) for risk of recurrent MI, 9 studies (19, 25–28, 30, 33, 36, 40) for risk of MACE, and three studies (29, 30, 39) for risk of repeat revascularization.

### Long-Term Effect of β-Blocker Use on All-Cause Mortality in Patients After MI

**Figure 1:**
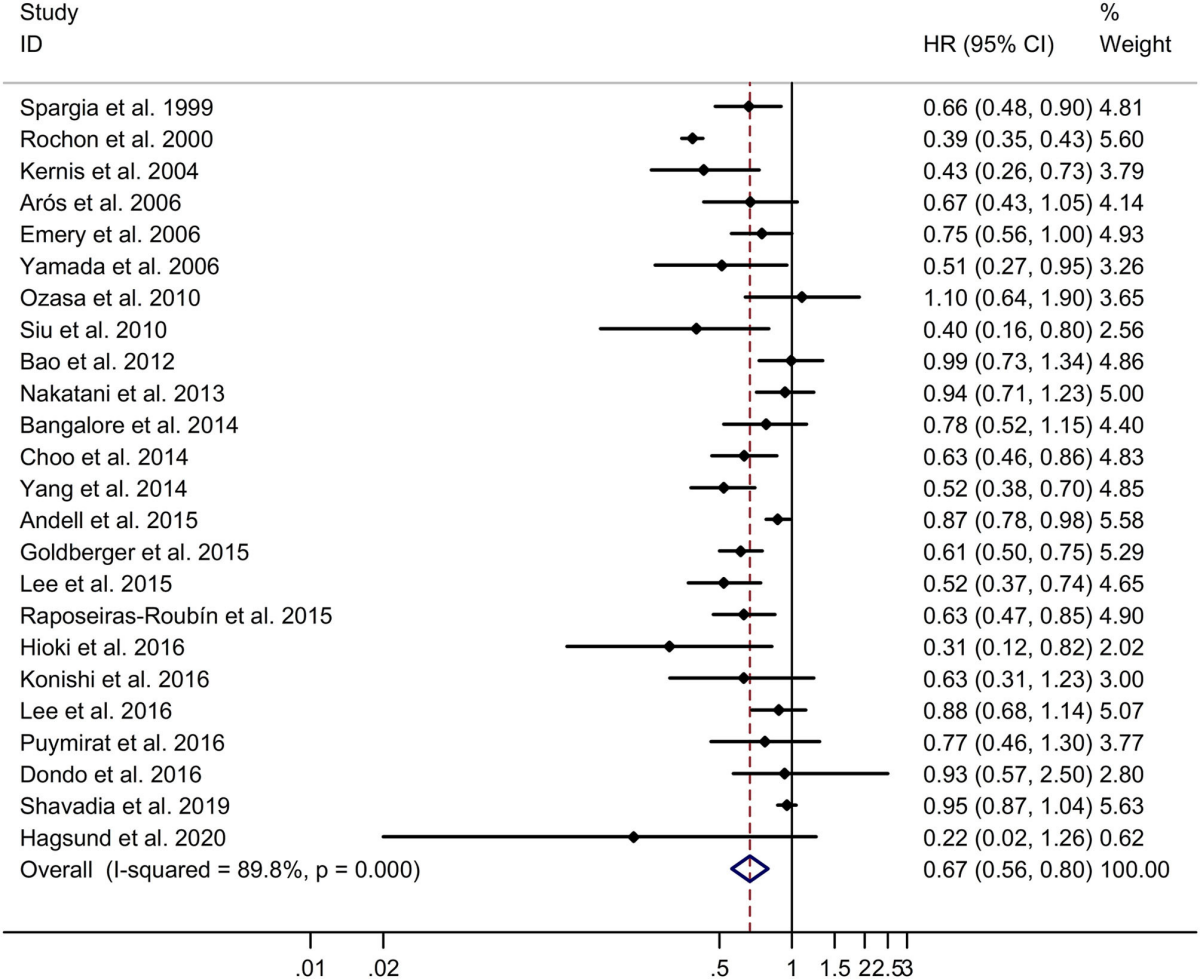
Forest plots exploring the long-term effect of β-blocker use on all-cause mortality in patients after MI. CIs, confidence intervals; HRs, hazard ratios; MI, myocardial infarction.

This study indicated that β-blocker group had significantly lower long-term all-cause mortality in post-MI patients, compared to no β-blocker group with a random effects model (HR, 0.67; 95% CI: 0.56–0.80, I^2^ = 89.8%, *p* < 0.001; Figure 1). Subgroup analyses showed that β-blocker group had significantly lower long-term all-cause mortality in post-MI patients, compared to no β-blocker group in both retrospective and prospective studies (retrospective studies: HR, 0.71; 95% CI: 0.62–0.82; prospective studies: HR, 0.61; 95% CI: 0.41–0.91; Supplementary Figure S2A). In addition, subgroup analyses showed that β-blocker group had significantly lower long-term all-cause mortality in post-MI patients, compared to no β-blocker group in both Caucasian and Asian populations (Caucasian populations: HR, 0.62; 95% CI: 0.50–0.76; Asian populations: HR, 0.69; 95% CI: 0.56–0.84; Supplementary Figure S3). Meta-regression analyses showed that history of HF was responsible for heterogeneity across studies regarding the long-term effect of β-blocker use on all-cause mortality in patients after MI (history of HF: *p* = 0.048). Sensitivity analyses indicated no change in the direction of effect when any one study was eliminated (Supplementary Figure S4A). In addition, Begg's test, Egger's test, and funnel plot showed no significant risk of publication bias (Begg's test: *p* = 0.172; Egger's test: *p* = 0.690; Supplementary Figures S5A, S6).

In addition, the study showed that the β-blocker group showed significantly lower long-term all-cause mortality in post-MI patients with low EF, compared to no β-blocker group with a random effects model (HR, 0.69; 95% CI: 0.59–0.81, I^2^ = 52.3%, *p* = 0.008; Supplementary Figure 7A).

### Long-Term Effect of β-Blocker Use on Cardiovascular Mortality in Patients After MI

**Figure 2:**
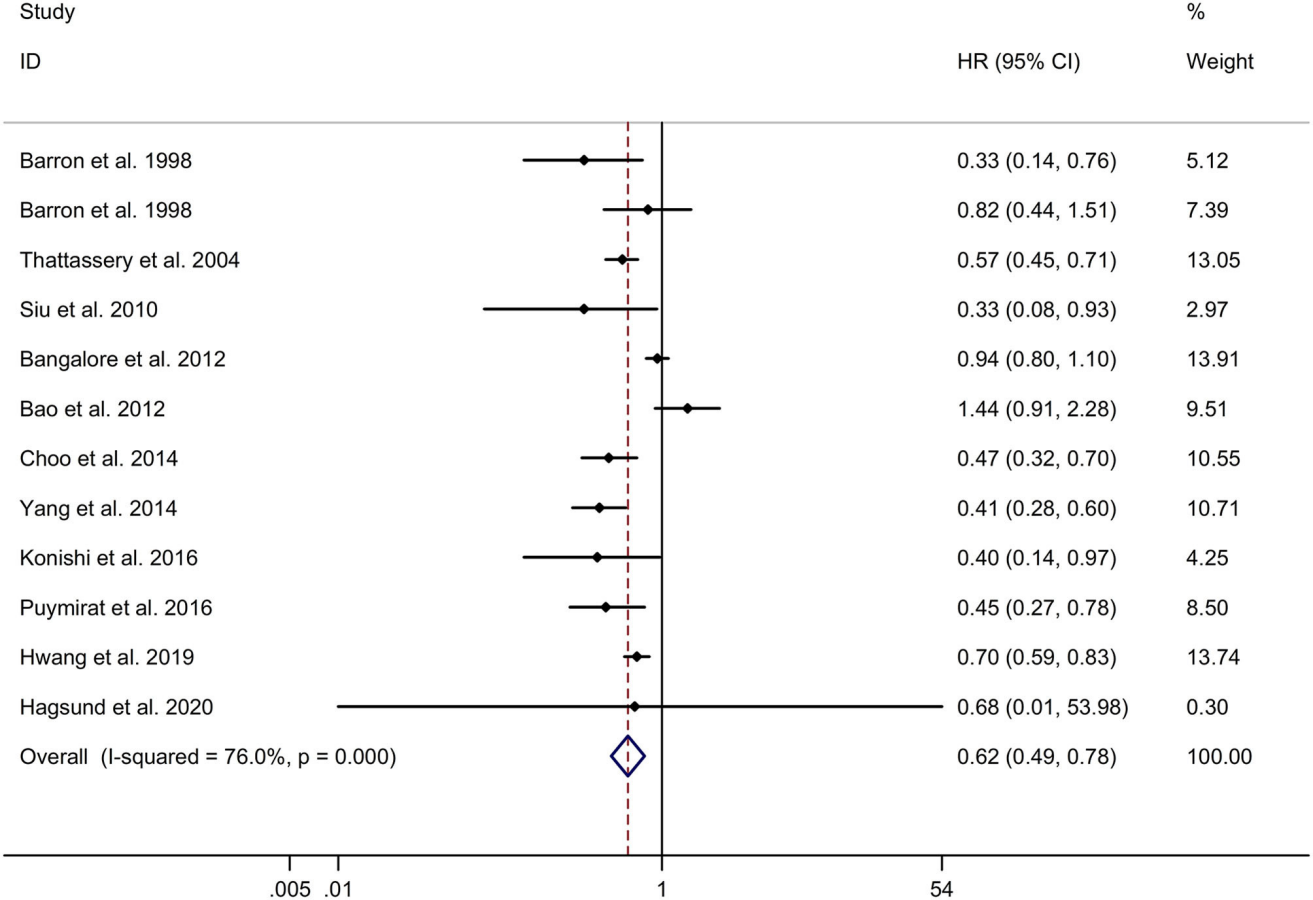
Forest plots exploring the long-term effect of β-blocker use on cardiovascular mortality in patients after MI. CIs, confidence intervals; HRs, hazard ratios; MI, myocardial infarction.

This study indicated that the β-blocker group showed significantly lower long-term cardiovascular mortality in post-MI patients, compared to no β-blocker group with a random effects model (HR, 0.62; 95% CI: 0.49–0.78, I^2^ = 76.0%, *p* < 0.001; Figure 2). Subgroup analyses showed that β-blocker group had significantly lower long-term cardiovascular mortality in post-MI patients, compared to no β-blocker group in both retrospective and prospective studies (retrospective studies: HR, 0.61; 95% CI: 0.45–0.84; prospective studies: HR, 0.60; 95% CI: 0.40–0.91; Supplementary Figure S2B). In addition, subgroup analyses showed that the β-blocker group had significantly lower long-term cardiovascular mortality in post-MI patients, compared to no β-blocker group in both Caucasian and Asian populations (Caucasian populations: HR, 0.63; 95% CI: 0.44–0.89; Asian populations: HR, 0.60; 95% CI: 0.41–0.88; Supplementary Figure S3B). Meta-regression analyses showed that no variables were responsible for heterogeneity across studies regarding the long-term effect of β-blocker use on cardiovascular mortality in patients after MI (all *p* > 0.05). Sensitivity analyses indicated no change in the direction of effect when any one study was eliminated (Supplementary Figure S4B). In addition, Begg's test, Egger's test, and funnel plot showed no significant risk of publication bias (Begg's test: *p* = 0.891; Egger's test: *p* = 0.176; Supplementary Figure 5B).

In addition, the study indicated that β-blocker group showed significantly lower long-term cardiovascular mortality in post-MI patients with low EF, compared to no β-blocker group with a random effects model (HR, 0.65; 95% CI: 0.48–0.87, I^2^ = 80.8%, *p* < 0.001; Supplementary Figure 7B).

### Long-Term Effect of β-Blocker Use on Risk of Hospitalization for HF in Patients After MI

**Figure 3:**
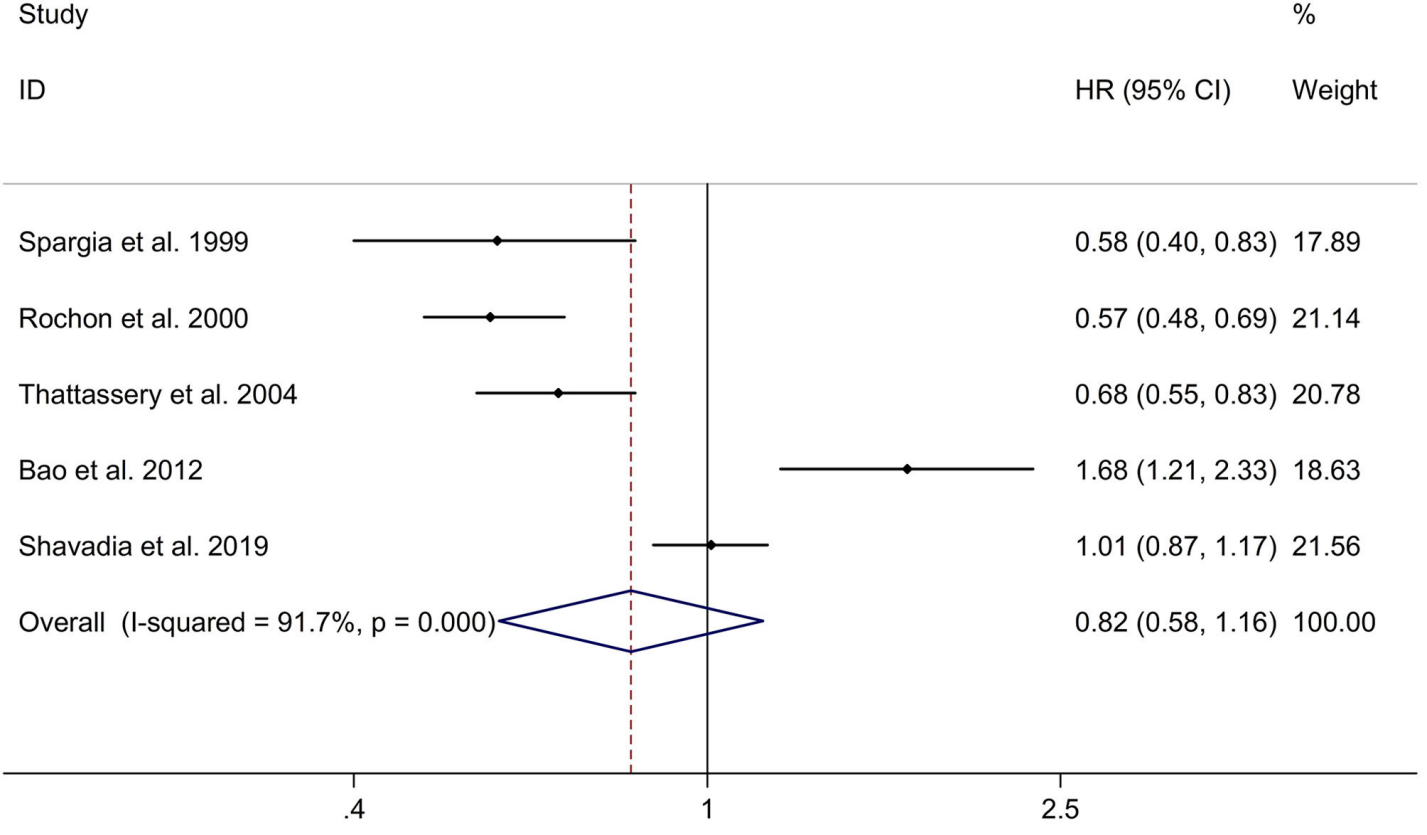
Forest plots exploring the long-term effect of β-blocker use on risk of hospitalization for HF in patients after MI. CIs, confidence intervals; HF, heart failure; HRs, hazard ratios; MI, myocardial infarction.

This study indicated no significant long-term effect of β-blocker use on risk of hospitalization for HF in post-MI patients with a random effects model (HR, 0.82; 95% CI: 0.58–1.16, I^2^ = 91.7%, *p* < 0.001; Figure 3). Subgroup analyses showed no significant long-term effect of β-blocker use on risk of hospitalization for HF in post-MI patients in retrospective studies (HR, 0.90; 95% CI: 0.62–1.31; Supplementary Figure S2C). However, subgroup analyses showed a significant long-term effect of β-blocker use on risk of hospitalization for HF in post-MI patients in Caucasian populations (HR, 0.70; 95% CI: 0.51–0.95; Supplementary Figure S3C). Meta-regression analyses showed that no variables were responsible for heterogeneity across studies regarding long-term effect of β-blocker use on risk of hospitalization for HF in post-MI patients (all *p* > 0.05). Sensitivity analyses indicated no change in the direction of effect when any one study was eliminated (Supplementary Figure S4C). In addition, Begg's test, Egger's test, and funnel plot showed no significant risk of publication bias (Begg's test: *p* = 1.000; Egger's test: *p* = 0.946; Supplementary Figure S5C).

### Long-Term Effect of β-Blocker Use on Risk of Recurrent MI in Patients After MI

**Figure 4:**
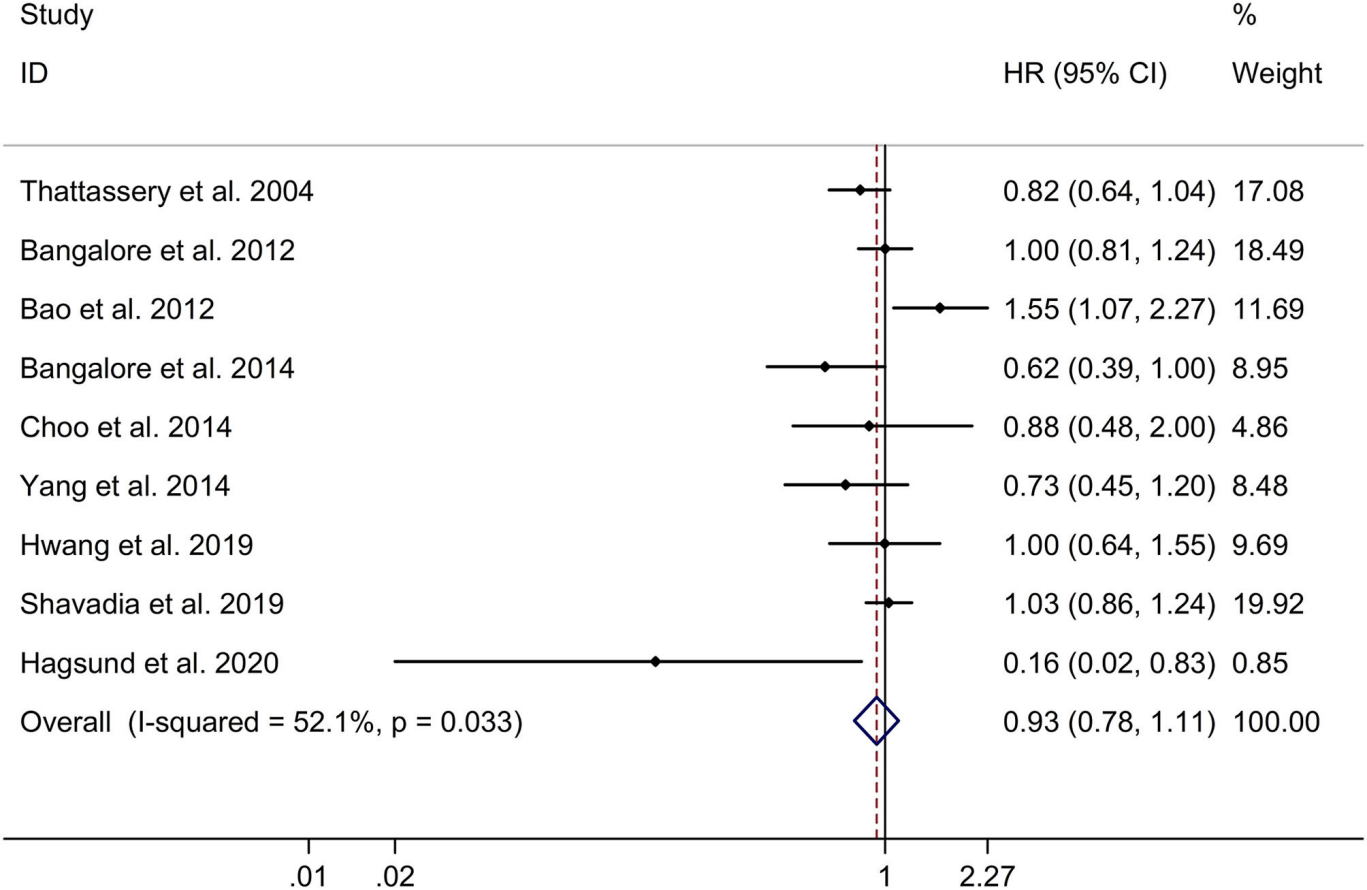
Forest plots exploring the long-term effect of β-blocker use on risk of recurrent MI in patients after MI. CIs, confidence intervals; HRs, hazard ratios; MI, myocardial infarction.

This study indicated no significant long-term effect of β-blocker use on risk of recurrent MI in post-MI patients with a random effects model (HR, 0.93; 95% CI: 0.78–1.11, I^2^ = 52.1%, *p* = 0.033; Figure 4). Subgroup analyses showed no significant long-term effect of β-blocker use on risk of recurrent MI in retrospective studies (HR, 0.92; 95% CI: 0.76–1.12; Supplementary Figure S2D). In addition, subgroup analyses showed no significant long-term effect of β-blocker use on risk of recurrent MI in post-MI patients in both Caucasian and Asian populations (Caucasian populations: HR, 0.81; 95% CI: 0.61–1.07; Asian populations: HR, 1.05; 95% CI: 0.84–1.31; Supplementary Figure S3D). Meta-regression analyses showed that no variables were responsible for heterogeneity across studies regarding long-term effect of β-blocker use on risk of recurrent MI in post-MI patients (all *p* > 0.05). Sensitivity analyses indicated no change in the direction of effect when any one study was eliminated (Supplementary Figure S4D). In addition, Begg's test, Egger's test, and funnel plot showed no significant risk of publication bias (Begg's test: *p* = 0.061; Egger's test: *p* = 0.235; Supplementary Figure S5D).

In addition, the study indicated that the β-blocker group showed no significant long-term effect of β-blocker use on risk of recurrent MI in post-MI patients with low EF, compared to no β-blocker group with a random effects model (HR, 0.92; 95% CI: 0.70–1.20, I^2^ = 58.6%, *p* = 0.025; Supplementary Figure S7C).

### Long-Term Effect of β-Blocker Use on Risk of MACE in Patients After MI

**Figure 5:**
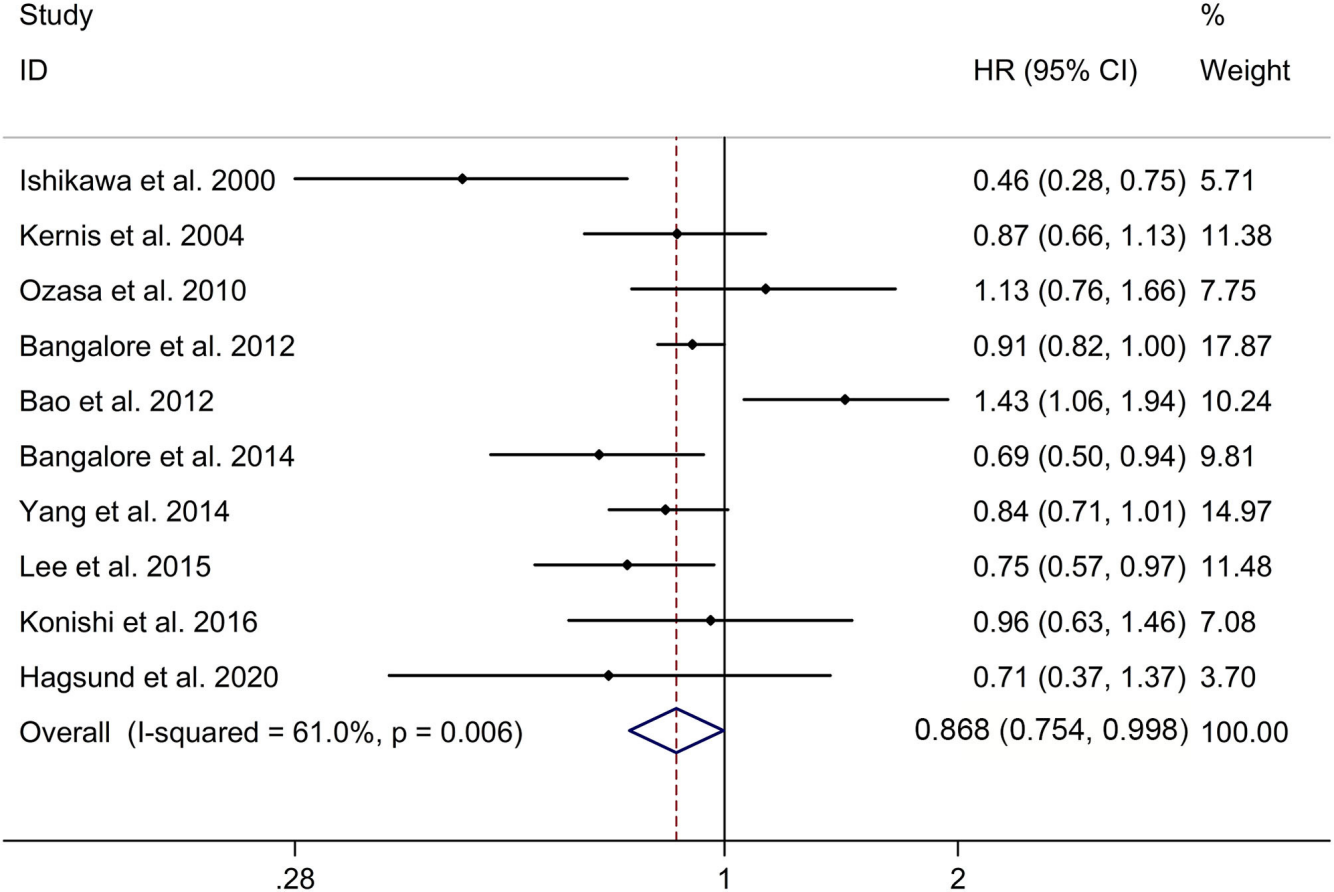
Forest plots exploring the long-term effect of β-blocker use on risk of MACE in patients after MI. CIs, confidence intervals; HRs, hazard ratios; MACE, major adverse cardiac events; MI, myocardial infarction.

This study showed a significant long-term effect of β-blocker use on risk of MACE in post-MI patients with a random effects model (HR, 0.868; 95% CI: 0.754–0.998, I^2^ = 61.0%, *p* = 0.006; Figure 5). Subgroup analyses showed a significant long-term effect of β-blocker use on risk of MACE in post-MI patients in Caucasian populations, but not in Asian populations (Caucasian populations: HR, 0.88; 95% CI: 0.79–0.97; Asian populations: HR, 0.89; 95% CI: 0.69–1.15; Supplementary Figure S3E). Meta-regression analyses showed that no variables were responsible for heterogeneity across studies regarding the long-term effect of β-blocker use on risk of MACE in post-MI patients (all *p* > 0.05). Sensitivity analyses indicated no change in the direction of effect when any one study was eliminated (Supplementary Figure S4E). In addition, Begg's test, Egger's test, and funnel plot showed no significant risk of publication bias (Begg's test: *p* = 0.421; Egger's test: *p* = 0.595; Supplementary Figure S5E).

In addition, the study indicated that β-blocker group showed no significant long-term effect of β-blocker use on risk of MACE in post-MI patients with low EF, compared to no β-blocker group with a random effects model (HR, 0.90; 95% CI: 0.79–1.02, I^2^ = 50.4%, *p* = 0.040; Supplementary Figure S7D).

### Long-Term Effect of β-Blocker Use on Risk of Stroke in Patients After MI

**Figure 6:**
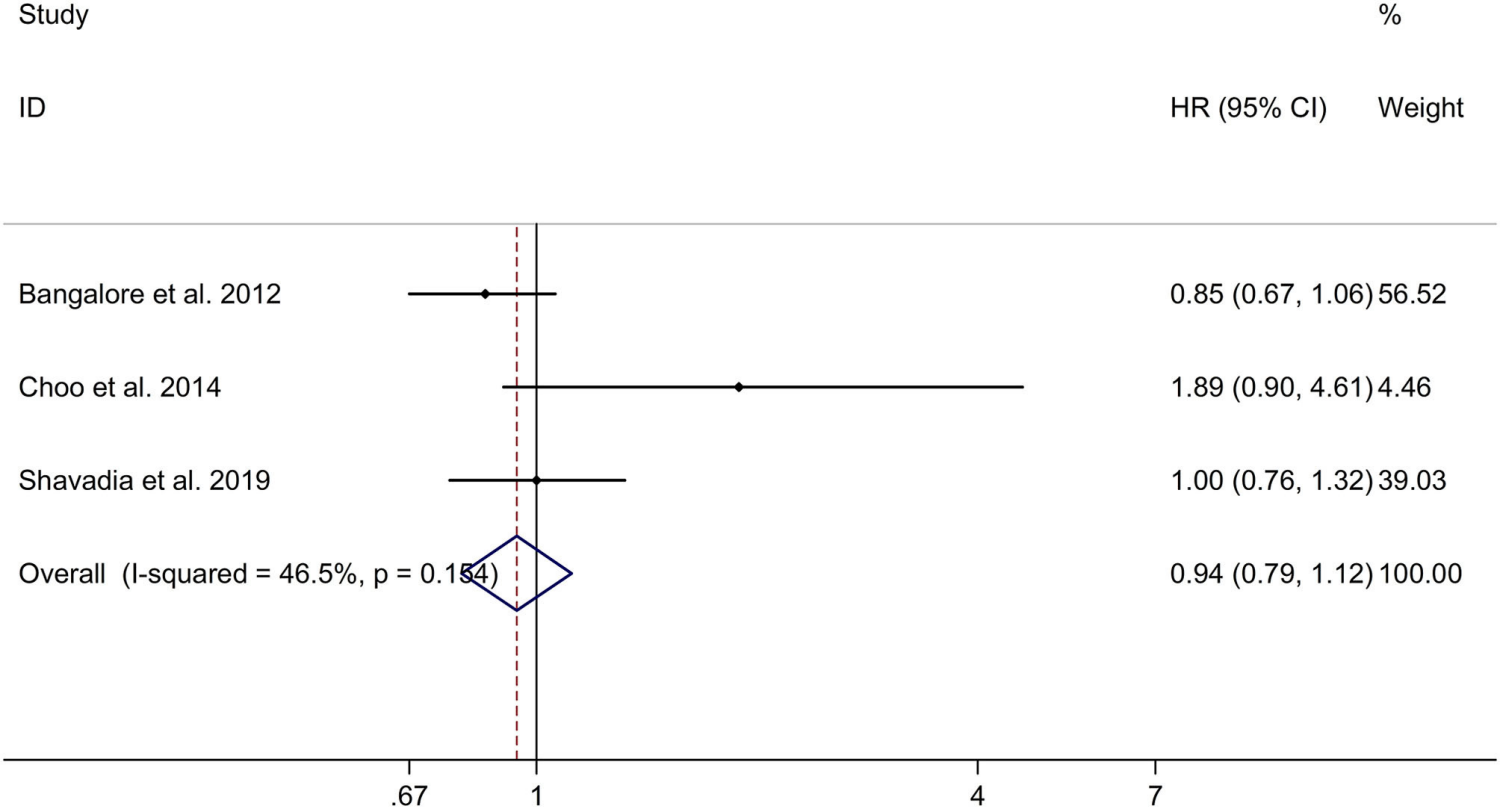
Forest plots exploring the long-term effect of β*-*blocker use on risk of stroke in patients after MI. CIs, confidence intervals; HRs, hazard ratios; MI, myocardial infarction.

This study showed no significant long-term effect of β-blocker use on risk of stroke in post-MI patients with a fixed effects model (HR, 0.94; 95% CI: 0.79–1.12, I^2^ = 46.5%, *p* = 0.154; Figure 6). Meta-regression analyses showed that no variables were responsible for heterogeneity across studies regarding long-term effect of β-blocker use on risk of stroke in post-MI patients (all *p* > 0.05). Sensitivity analyses indicated no change in the direction of effect when any one study was eliminated (Supplementary Figure S4F). In addition, Begg's test, Egger's test, and funnel plot showed no significant risk of publication bias (Begg's test: *p* = 0.117; Egger's test: *p* = 0.183; Supplementary Figure S5F).

### Long-Term Effect of β-Blocker Use on Risk of Repeat Revascularization in Patients After MI

**Figure 7:**
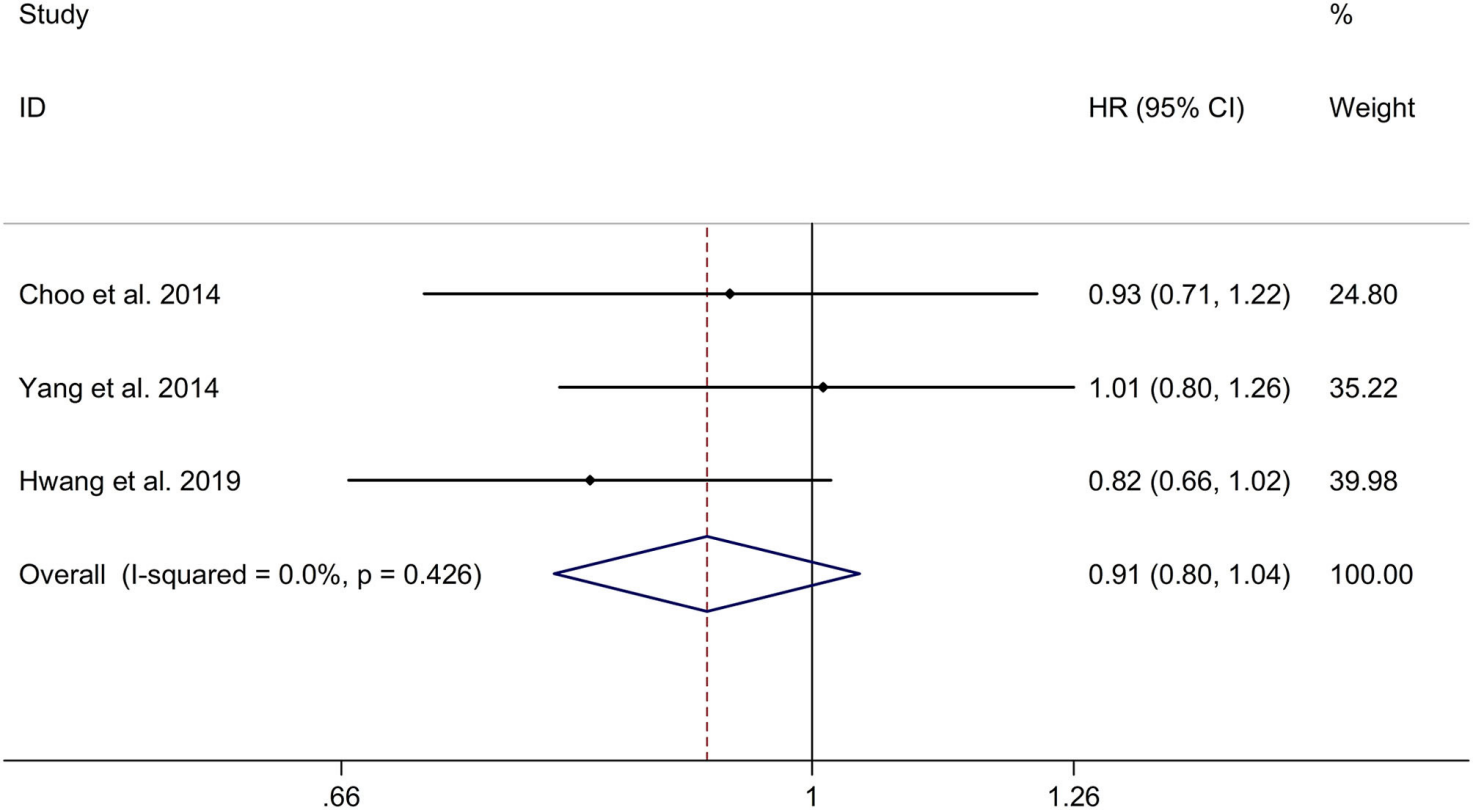
Forest plots exploring the long-term effect of β-blocker use on risk of repeat revascularization in patients after MI. CIs, confidence intervals; HRs, hazard ratios; MI, myocardial infarction.

This study showed no significant long-term effect of β-blocker use on risk of repeat revascularization in post-MI patients with a fixed effects model (HR, 0.91; 95% CI: 0.80–1.04, I^2^ = 0.0%, *p* = 0.426; Figure 7). Meta-regression analyses showed that no variables were responsible for heterogeneity across studies regarding long-term effect of β-blocker use on risk of repeat revascularization in post-MI patients (all *p* > 0.05). Sensitivity analyses indicated no change in the direction of effect when any one study was eliminated (Supplementary Figure S4G). In addition, Begg's test, Egger's test, and funnel plot showed no significant risk of publication bias (Begg's test: *p* = 0.602; Egger's test: *p* = 0.747; Supplementary Figure S5G).

In addition, the study indicated that the β-blocker group showed no significant long-term effect of β-blocker use on risk of repeat revascularization in post-MI patients with low EF, compared to no β-blocker group with a fixed effects model (HR, 0.91; 95% CI: 0.80–1.04, I^2^ = 0.0%, *p* = 0.426; Supplementary Figure S7E).

### Meta-Regression Results and Risk of Bias

Supplementary Table S2 shows results of meta-regression studies. Supplementary Table S3 contained full assessment. The assessment of the 29 observational studies was performed with the Newcastle–Ottawa quality assessment scale (on the study and outcome level) and showed that the average quality was good (6.79, moderate risk of bias).

## Discussion

The present meta-analysis showed significant long-term effects of β-blocker use on all-cause mortality, cardiovascular mortality, and risk of MACE in post-MI patients, whereas no significant long-term effect was shown on risk of hospitalization for HF, risk of recurrent MI, risk of stroke, and risk of repeat revascularization in post-MI patients.

The present meta-analysis showed a significant long-term effect of β-blocker use on all-cause mortality in post-MI patients. The result was not corresponding to the previous meta-analysis (14). This study included more studies, compared to the previous meta-analysis. The meta-analysis published in 2019 included studies published after January 1, 2000. In addition, the meta-analysis published in 2019 included studies where none or only a minority of patients had a history of HF, were in Killip class≥ III, or had LVEF < 40% at baseline. However, this study included all studies exploring the long-term effect of β-blocker use on all-cause mortality in post-MI patients. Limited inclusion criteria might be the source of a significant publication bias in the meta-analysis published in 2019. In addition, meta-regression analysis in the present meta-analysis showed that the history of HF was responsible for heterogeneity across studies regarding the long-term effect of β-blocker use on all-cause mortality in patients after MI. Regarding the impact of HF on the long-term effect of β-blocker use on clinical outcomes in post-MI patients, Rochon et al. (11) reported that β-blocker use is associated with improved clinical outcomes in patients after MI with a history of HF, whereas Dondo et al. (38) reported that among post-MI patients who did not have a history of HF, β-blocker use was not associated with a lower risk of death at time point up to 1 year. Regarding the impact of LVEF on the long-term effect of β-blocker use on clinical outcomes in post-MI patients, Lee et al. (33) reported that β-blocker use has beneficial clinical outcomes in the era of primary PCI for STEMI, regardless of the LVEF. However, Kernis et al. (19) found that β-blocker therapy after successful primary PCI is associated with a decreased six-month mortality, with the greatest benefit in patients with a low ejection fraction. Ozasa et al. (25) reported that β-blocker use was not associated with better long-term clinical outcomes in patients with STEMI who underwent primary PCI and had preserved LVEF. Thus, regarding the impact of β-blocker dose on the long-term effect of β-blocker use on clinical outcomes in post-MI patients, Hwang et al. (39) found that there was no significant additional benefit of high-dose β-blocker compared to low-dose β-blockers in 1-year risk of cardiovascular mortality in post-MI patients. Shavadia et al. (13) found that β-blocker use beyond 3 years post-MI, regardless of the dose achieved, was not associated with better clinical outcomes.

The study was novel to compute results of studies exploring the long-term effect of β-blocker use on other clinical outcomes (including cardiovascular mortality, risk of hospitalization for HF, risk of recurrent MI, risk of MACE, risk of stroke, and risk of repeat revascularization) in post-MI patients. However, limited numbers of studies were included to explore the long-term effect of β-blocker use on risk of hospitalization for HF, risk of recurrent MI, risk of MACE, risk of stroke, and risk of repeat revascularization in post-MI patients, especially on risk of stroke and risk of repeat revascularization. Thus, more large-scale prospective studies were essential to explore the long-term effect of β-blocker use on these clinical outcomes. Regarding the long-term effect of β-blocker use on risk of MACE, subgroup analyses showed a significant long-term effect of β-blocker use on risk of MACE in Caucasians but not in the Asian population. The positive effects of β-blocker use can be offset more in the Asian population than in the Caucasian population due to the susceptibility of the Asian population to the adverse effects of β-blockers. Previous studies supported more frequent coronary artery vasospasms and a more sensitive response of heart rate and blood pressure in the Asian population with lower doses of β-blockers (41–43), which may be due to differences in β1-receptor sensitivity between Asians and Westerners (42, 44).

There were some limitations in this meta-analysis. First, HRs used in this study were adjusted HRs. Adjusted covariates cover measured confounding variables, but they could not account for unmeasured variables. Second, due to the limited number of included studies, more large-scale prospective studies were essential to explore the long-term effect of β-blocker use on these clinical outcomes. Third, because many kinds of beta-blockers were included in some articles, it is difficult to perform a subanalysis or meta-regression study based on the type and amount of beta-blocker to confirm whether the heterogeneity of the results is caused by the type and amount of β-blockers. Fourth, due to high heterogeneity of this analysis, this study may suffer from confounding and should be interpreted as an observational association rather than a causal relationship.

## Conclusions

In conclusion, the present meta-analysis demonstrated significant long-term effects of β-blocker use on all-cause mortality, cardiovascular mortality, and risk of MACE in post-MI patients, whereas no significant long-term effect was shown on risk of hospitalization for HF, risk of recurrent MI, risk of stroke, and risk of repeat revascularization in post-MI patients. More large-scale prospective studies were essential to explore the long-term effect of β-blocker use on these clinical outcomes.

## Data Availability Statement

The original contributions presented in the study are included in the article/Supplementary Material, further inquiries can be directed to the corresponding author.

## Author Contributions

CL contributed to conceptualization, methodology, software, and writing—original draft. CZ contributed to software, validation, formal analysis, and data curation. SG contributed to validation, formal analysis, and data curation. XC contributed to validation and formal analysis. ZT contributed to conceptualization, methodology, writing, reviewing, editing, and supervision. All authors contributed to the article and approved the submitted version.

## Conflict of Interest

The authors declare that the research was conducted in the absence of any commercial or financial relationships that could be construed as a potential conflict of interest.

## Publisher's Note

All claims expressed in this article are solely those of the authors and do not necessarily represent those of their affiliated organizations, or those of the publisher, the editors and the reviewers. Any product that may be evaluated in this article, or claim that may be made by its manufacturer, is not guaranteed or endorsed by the publisher.
