# Supplementary material for: Long-Term Effect of β-Blocker Use on Clinical Outcomes in Postmyocardial Infarction Patients: A Systematic Review and Meta-Analysis
Source: Front Cardiovasc Med. 2022 Apr 8;9:779462. doi: 10.3389/fcvm.2022.779462 (PMC9024047; doi:10.3389/fcvm.2022.779462)
Supplement: Supplementary file 1 [file Table_1.docx]

| Supplementary table 1. Characteristics of included studies. | | | | | | | | | | | | | | | | | | | | |
| --- | --- | --- | --- | --- | --- | --- | --- | --- | --- | --- | --- | --- | --- | --- | --- | --- | --- | --- | --- | --- |
| Study/Year | Study type | Country | Sample size | Mean age  (years) | Gender (male%) | STEMI  (%) | type and daily dosage of the β-blocker | PCI  (%) | event count information | LVEF  (%) | History  of  HF (%) | Killip  ≤2 (%) | Hypertension  (%) | Diabetes  (%) | Smoking  (%) | Prior  MI  (%) | ARB/ACEI (%) | ASA  (%) | Statins  (%) | Follow-up  (years) |
| Barron et al. 1998 ^[^[^1^](#_ENREF_1)^]^ | Retrospective | USA | 176/189/654 | 62.0/ 58.7 | 72.2/73 | NR | propranolol hydrochloride, 160 mg/d; metoprolol tartrate, 200 mg/d; or atenolol, 100 mg/d | NR | cardiovascular deaths (n=145) in β blocker group | NR | NR | NR | NR | NR | 34.7/ 41.8 | 22.7/ 22.8 | NR | 89.2/ 83.6 | NR | 2.0 |
| Spargia et al. 1999 ^[^[^2^](#_ENREF_2)^]^ | Retrospective | UK | 443/ 1543 | 63.1 (11.1) | 75.6 | NR | NR | NR | all cause mortality (52 deaths), hospitalization for HF (41 events) in β blocker group | 13.7 | 7.0 | NR | 28.7 | 11.3 | NR | 21.7 | NR | 85.1 | NR | 1.25 |
| Ishikawa et al. 2000 ^[^[^3^](#_ENREF_3)^]^ | Retrospective | Japan | 833/ 650 | 59.1 ± 10.5 | 78.6 | NR | metoprolol (40-120 mg / day) was administered to 432 patients, atenolol (50 mg / day) to 86 patients, bisoprolol (5 mg / day) to 85 patients, propranolol (60-120 mg / day) to 64 patients and nipradilol (6-12 mg / day) to 55 patients. The remaining 111 patients were administered either carteolol (10-20 mg / day: 18 patients), celiprolol (100-200 mg /day:14 patients), nadolol (30-60 mg / day: 13 patients), arotinolol (20-30 mg /day: 13 patients) or betaxolol (5-10 mg / day: 11 patients) | NR | MACE (27) in β blocker group | NR | NR | NR | NR | NR | NR | NR | NR | NR | NR | 1.45 |
| Rochon et al. 2000 ^[^[^4^](#_ENREF_4)^]^ | Prospective | Canada | 2248/ 3068/758/7549 | 74.8 (6.2)/ 73.6 (5.8)/ 73.1 (5.5) | 55.5/56.8/57.5 | NR | Atenolol, Metoprolol, Propranolol, Timolol, Acebutolol,  Labetalol, Nadolol, Pindolol,  Sotalol, Low, Standard and high doses | NR | all cause mortality (452 deaths), HF (2534 events) in β blocker group | NR | 27.3/20.4/20.4 | NR | NR | 15.5/17.5/16.9 | NR | NR | 46.6/ 40.2/ 43.8 | 81.6/ 83.6/ 85.5 | NR | 1 |
| Kernis et al. 2004 ^[^[^5^](#_ENREF_5)^]^ | Retrospective | USA/Europe | 1,661/781 | 60 ± 12 | 75 | 100 | NR | 100 | Mortality (37 deaths), MACE (233 events) in β blocker group | 48.9 | 2.3 | 98.7 | 44.9 | 16.6 | 66.2 | 13.8 | NR | NR | NR | 0.5 |
| Thattassery et al. 2004 ^[^[^6^](#_ENREF_6)^]^ | Retrospective | USA | 789/1442 | NR | NR | NR | The initial dose of carvedilol should be 6.25 mg bid, which can be reduced in half if not tolerated. This dose can be increased every 3 to 10 days if heart rate > 50 and blood pressure > 80 mmHg. Over a period of 4–6 weeks, patients should be titrated to a maximum of 25 mg bid. With gradual titration in this man-  ner, 74% of patients in CAPRICORN achieved  maximum dosing, 11% achieved 12.5 mg bid  dosing, and 7% achieved 6.25 mg bid dosing | NR | cardiovascular deaths (n=56), HF (130 events), recurrent MI (97 events) in β blocker group | NR | NR | NR | NR | NR | NR | NR | NR | NR | NR | 1.3 |
| Arós et al. 2006 ^[^[^7^](#_ENREF_7)^]^ | Prospective | USA | Total 5397 | NR | NR | NR | NR | NR | NR | NR | NR | NR | NR | NR | NR | NR | NR | NR | NR | 1.0 |
| Emery et al. 2006 ^[^[^8^](#_ENREF_8)^]^ | Retrospective | Europe | 5422/1684 | 66 | 68 | 0 | carvedilol | 15 | Mortality (95) in β blocker group | NR | NR | NR | 62 | 25 | 57 | 28 | 26 | 41 | 25 | 0.5 |
| Yamada et al. 2006 ^[^[^9^](#_ENREF_9)^]^ | Prospective | Japan | 400/ 146 | 62 ± 11 | 75 | 82.5 | metoprolol  (80%) followed by propranolol (11%) and atenolol (6%) | 61.1 | NR | 54.0 | NR | 87.7 | 41.3 | 37.4 | 64.3 | 0 | 51.6 | 92.1 | 31.9 | 2.0 |
| Ozasa et al. 2010 ^[^[^10^](#_ENREF_10)^]^ | Retrospective | Japan | 349/561 | 66.4 ± 11.5 | 76.0 | 100 | NR | 100 | Mortality (23), MACE (39) in β blocker group | 52.3 | 17.0 | NR | 68.0 | 38.0 | 38.0 | 8 | 76.2 | 99.1 | 54.6 | 3.0 |
| Siu et al. 2010 ^[^[^11^](#_ENREF_11)^]^ | Retrospective | China | 154/54 | 62 ±1 | 72 | NR | 125 patients on β-blocker (81%) received metoprolol at mean dose of 64 ± 3.3 mg/day, 15 patients (10%) received  atenolol at 61 ± 7.2 mg/day, 12 patients (8%) on carvedilol at 9.4 ± 1.8 mg/day, and two patients (1%) on bisoprolol 6.25±3.8mg/day | 27 | Mortality (14), cardiac death in β blocker group | 55 | NR | NR | 90 | 58 | 50 | NR | 100 | 100 | 81 | 4.87 |
| Bangalore et al. 2012 ^[^[^12^](#_ENREF_12)^]^ | Retrospective | USA/Europe | 3379/3379 | 68.67 (9.99) | 75.1 | NR | Carvedilol; atenolol | NR | NR | NR | 22.3 | NR | 73.6 | 37.3 | 9.7 | NR | 69.4 | 75.8 | 74.5 | 3.6 |
| Bao et al. 2012 ^[^[^13^](#_ENREF_13)^]^ | Retrospective | Japan | 1,614/ 2,078 | 65.8 ± 12.2 | 74.6 | 100 | NR | 100 | All-cause death (29), Cardiac death (19), MI (11), Hospitalization for HF (34) in β blocker group | 53.5 | 27.3 | NR | 78.7 | 31.4 | 41.6 | 8.6 | 75.7 | 99.5 | 56.6 | 2.6 |
| Nakatani et al. 2013 ^[^[^14^](#_ENREF_14)^]^ | Retrospective | Japan | 1,923/ 1,923 | 64.4 ± 11.4 | 77.8% | 100 | carvedilol, metoprolol, bisoprolol, atenolol, and other BBs | 100 | All-cause death (150) in β blocker group | NR | NR | 84.6 | 32.4 | 61.0 | 64.9 | 10.9 | 77.1 | 95.5 | 42.6 | 3.9 |
| Bangalore et al. 2014 ^[^[^15^](#_ENREF_15)^]^ | Retrospective | USA/Europe | 981/981 | 64.5±9.14 | 79.4 | NR | atenolol or metoprolol | NR | NR | NR | 0 | NR | 69.7 | 35.4 | 18.2 | NR | 17.7 | 98.0 | 80.4 | 2.3 |
| Choo et al. 2014 ^[^[^16^](#_ENREF_16)^]^ | Retrospective | Korea | 2424/595 | 60.9±12.1 | 73.2 | 58.1 | Carvedilol, bisoprolol, atenolol, or other β-blockers | 100 | cardiac death (184), stroke (61), MI (36), revascularization (318) in β blocker group | 60.4 | NR | 93.8 | 50.0 | 40.6 | 44.0 | 3.3 | 81.8 | 99.7 | 90.4 | 3.0 |
| Yang et al. 2014 ^[^[^17^](#_ENREF_17)^]^ | Retrospective | Korea | 2,650/ 1,325 | 66 (55–74) | 73.0 | 100 | NR | 100 | all-cause death (144), cardiac death (76) in β blocker group | 50.0 | 0.9 | 85.3 | 43.6 | 25.3 | 45.7 | 6.5 | 75.9 | 98.6 | 80.8 | 1.0 |
| Andell et al. 2015 ^[^[^18^](#_ENREF_18)^]^ | Prospective | Sweden | 4086/772 | 74 (67 to 80) | 56.3 | NR | NR | 4.3 | NR | NR | 19.9 | NR | 54.5 | 22.3 | 39.6 | 19.8 | 31.9 | 38.8 | 22.0 | 2.83 |
| Goldberger et al. 2015 ^[^[^19^](#_ENREF_19)^]^ | Prospective | USA | 1,448 /2,247/1,541/809/567 | 64.5 ±13.6/62.6 ±13.6/64.0 ±13.3/64.2 ±13.1 | 67.1/69.2/68.5/70.5 | 49.6/44.6/42.3/36.7 | metoprolol 200 mg/day; carvedilol 50 mg/day (Coreg CR [GlaxoSmithKline Pharmaceuticals, Philadelphia, Pennsylvania]–equivalent dose 80 mg/day); propranolol 180 mg/day; timolol 20 mg/day; bisoprolol 10 mg/day;and atenolol 100mg/day | 66.5/63.6/55.7/63.8 | all-cause death (587) in β blocker group | NR | 11.5/8.0/9.7/13.8 | NR | 59.2/65.3/73.4/82.4 | 27.6/30.7/34.6/41.6 | 33.9/36.2/32.0/26.5 | 17.3/18.3/24.6/26.9 | 62.1/ 68.6/ 70.3/ 71.8 | 93.4/ 93.4/ 94.1/ 92.1 | 86.2/ 89.2/ 86.4/ 89.7 | 3 |
| Lee et al. 2015 ^[^[^20^](#_ENREF_20)^]^ | Retrospective | Korea | 598/303 | 56 ± 12 | 79.5 | 100 | Carvedilol was used most frequently in 277 patients  (46.3% in the β-blocker group) and metoprolol in 268 patients (44.8%) | 100 | NR | 51.7 | NR | 87.9 | 40.1 | 21.9 | 64.8 | 3.2 | 93.2 | 99.2 | 66.3 | 4.5 |
| Raposeiras-Roubín et al. 2015 ^[^[^21^](#_ENREF_21)^]^ | Retrospective | Spain | 555/555 | 66.1 (12.1) | 69.0 | 28.0 | Metoprolol | 65.2 | all-cause death (264) in β blocker group | NR | 10.8 | NR | 56.7 | 25.9 | 27.6 | 9 | 58.9 | 87.2 | 82.8 | 5.2 |
| Hioki et al. 2016 ^[^[^22^](#_ENREF_22)^]^ | Retrospective | Japan | 251/193 | 65.2 ± 11.7 | 81.8 | 81.8 | carvedilol (92.8 %), bisoprolol (4.0 %), and others  (3.2 %) | 100 | all-cause death (7) in β blocker group | 56.1 | NR | 100 | 22.7 | 24.1 | 65.1 | NR | 85.1 | NR | 100 | 2.9 |
| Konishi et al. 2016 ^[^[^23^](#_ENREF_23)^]^ | Retrospective | Japan | 103/103 | 64.3 ± 11.8 | 80.6 | 100 | NR | 100 | Cardiac death (6), MACE (12), all-cause death (2) in β blocker group | 56.4 | 0 | NR | 60.2 | 38.9 | 35.0 | 0 | 81.5 | 99.0 | 50.5 | 4.7 |
| Lee et al. 2016 ^[^[^24^](#_ENREF_24)^]^ | Retrospective | Korea | 3683/3578 | 61.9 ±12.8 | 75.4 | NR | Carvedilol (73.7%) was the predominantly prescribed BB, followed by nebivolol (9.3%) and bisoprolol (7.8%) | 100 | all-cause death (1080) in β blocker group | NR | 2.1 | NR | 30.4 | 27.1 | NR | 0 | 0 | 95.2 | 94.3 | 2.4 |
| Puymirat et al. 2016 ^[^[^25^](#_ENREF_25)^]^ | Prospective | France | 1783/434 | 64.4 | 72.0 | 56.0 | Metoprolol | 48.7 | all-cause death (70), cardiac death (53) in β blocker group | 55.0 | 0 | 100 | 54.8 | 31.8 | 32.5 | 14.5 | 37.5 | NR | 32.2 | 1.0 |
| Dondo et al. 2016 ^[^[^26^](#_ENREF_26)^]^ | Retrospective | UK | 141,097/ 7,217 | 63.3±13.4 | 71.4 | 53.0 | NR | 45.9 | all-cause death (6914) in β blocker group | NR | 0 | NR | 36.4 | 11.6 | 65.7 | 0 | 88.3 | 96.7 | 96.3 | 1.0 |
| Hwang et al. 2019 ^[^[^27^](#_ENREF_27)^]^ | Prospective | Korea | 8,258/ 1,636/ 2,015 | 63.3±12.4/ 62.5±12.7 | 74.7/ 75.1 | 50.3/ 44.1 | metoprolol was defined as 200 mg/day | 93.6/ 89.5 | cardiac death (257) in β blocker group | NR | 1.3/ 2.0 | 89.3/89.9 | 49.3/ 62.2 | 27.6/ 33.7 | NR | 6.4/ 13.1 | 84.9/ 84.7 | 99.9/ 100 | 95.2/ 93.8 | 1.0 |
| Shavadia et al. 2019 ^[^[^28^](#_ENREF_28)^]^ | Retrospective | USA | 4980/ 1913 | 75 (70-81) | 44.8 | 8.7 | metoprolol 200 mg/d; carvediolol 50 mg/d; bisoprolol  10 mg/d; atenolol 100 mg/d; propranolol 160 mg/d; labetalol  400 mg/d; nadolol 160 mg/d; nebivolol 10 mg/d; acebu-  tolol 400 mg/d; and pindolol 20 mg/d | 57.4 | NR | 22.8% < 0.4 | NR | NR | NR | 30.5 | 13.5 | 24.5 | 72.5 | NR | 84.4 | 3.0 |
| Hagsund et al. 2020 ^[^[^29^](#_ENREF_29)^]^ | Retrospective | Sweden | 206/ 141 | 64.1 ± 10 | 68.9 | 41.7 | NR | 82.5 | ,mortality (6), MACE (23) in β blocker group | 70% < 0.5 | 5.8 | NR | 52.4 | 24.3 | 37.9 | 18.9 | 72.3 | 90.8 | 99.5 | 1.0 |

Abbreviations: ACEI, angiotensin-converting enzyme inhibitor; ASA, acetylsalicylic acid; ARB, angiotensin receptor blocker; HF, heart failure; LVEF, left ventricular ejection fraction; MI, myocardial infarction; NR, not reported; PCI, percutaneous coronary intervention; STEMI, ST-elevation myocardial infarction; UK, United Kingdom; USA, United States of America.
