# Supplementary material for: Long-Term Effect of β-Blocker Use on Clinical Outcomes in Postmyocardial Infarction Patients: A Systematic Review and Meta-Analysis
Source: Front Cardiovasc Med. 2022 Apr 8;9:779462. doi: 10.3389/fcvm.2022.779462 (PMC9024047; doi:10.3389/fcvm.2022.779462)
Supplement: Supplementary file 2 [file Table_2.docx]

Supplementary table 2. Results of meta-regression studies.

| Indicators | all-cause mortality | cardiovascular mortality | risk of hospitalization for HF | risk of recurrent MI | risk of MACE | risk of stroke | risk of repeat revascularization |
| --- | --- | --- | --- | --- | --- | --- | --- |
| publication year | 0.919 | 0.936 | 0.136 | 0.899 | 0.492 | 0.982 | 0.436 |
| age | 0.056 | 0.774 | 0.942 | 0.400 | 0.294 | 0.557 | 0.687 |
| gender | 0.152 | 0.985 | 0.928 | 0.934 | 0.813 | 0.894 | 0.426 |
| ratio of STEMI | 0.411 | 0.963 | NA | 0.430 | 0.641 | NA | 0.446 |
| ratio of patients treated with PCI | 0.497 | 0.407 | NA | 0.396 | 0.487 | NA | 0.436 |
| LVEF | 0.900 | 0.805 | NA | 0.946 | 0.599 | NA | NA |
| ratio of history of HF | 0.048 | 0.149 | 0.467 | 0.128 | 0.140 | NA | NA |
| ratio of Killip class≤2 | 0.402 | 0.209 | NA | 0.662 | 0.813 | NA | 0.735 |
| ratio of history of hypertension | 0.577 | 0.204 | NA | 0.338 | 0.075 | NA | 0.491 |
| ratio of history of diabetes | 0.193 | 0.873 | 0.105 | 0.636 | 0.655 | 0.688 | 0.981 |
| ratio of history of smoking | 0.444 | 0.753 | NA | 0.424 | 0.109 | 0.310 | NA |
| ratio of prior MI | 0.487 | 0.728 | 0.464 | 0.381 | 0.583 | NA | 0.940 |
| ratio of treatment with ARBs/ACEI | 0.051 | 0.971 | 0.257 | 0.526 | 0.523 | 0.305 | 0.426 |
| ratio of treatment with ASA | 0.141 | 0.669 | 0.109 | 0.197 | 0.293 | NA | 0.446 |
| ratio of treatment with statins | 0.057 | 0.590 | NA | 0.256 | 0.052 | 0.393 | 0.427 |
| follow-up duration | 0.772 | 0.627 | 0.078 | 0.734 | 0.234 | 0.573 | 0.921 |

Abbreviation: ACEI, angiotensin-converting enzyme inhibitors; ARBs, angiotensin receptor blockers; ASA, acetylsalicylic acid; HF, heart failure; LVEF, left ventricular ejection fraction; MACE, major adverse cardiac event; MI, myocardial infarction; NA, not applicable; PCI, percutaneous coronary intervention; STEMI, ST-elevation myocardial infarction.
