# Supplementary material for: Long-Term Effect of β-Blocker Use on Clinical Outcomes in Postmyocardial Infarction Patients: A Systematic Review and Meta-Analysis
Source: Front Cardiovasc Med. 2022 Apr 8;9:779462. doi: 10.3389/fcvm.2022.779462 (PMC9024047; doi:10.3389/fcvm.2022.779462)
Supplement: Supplementary file 3 [file Table_3.docx]

Supplementary table 3. Quality assessment

| Study | Newcastle-Ottawa Scale | | | | | | | | | | |
| --- | --- | --- | --- | --- | --- | --- | --- | --- | --- | --- | --- |
|  | Selection | | | | Comparability | | | Outcome | | | Total |
|  | 1^a^ | 2^b^ | 3^c^ | 4^d^ | | 5^e^ | 6^f^ | | 7^g^ | 8^h^ |  |
| Case-control studies | | | | | | | | | | | |
| Barron et al. 1998 ^[^[^1^](#_ENREF_1)^]^ | 1 | 1 | 0 | 1 | | 1 | 1 | | 0 | 1 | 6 |
| Spargia et al. 1999 ^[^[^2^](#_ENREF_2)^]^ | 1 | 1 | 0 | 1 | | 1 | 0 | | 1 | 1 | 6 |
| Ishikawa et al. 2000 ^[^[^3^](#_ENREF_3)^]^ | 1 | 1 | 0 | 1 | | 0 | 1 | | 1 | 1 | 6 |
| Kernis et al. 2004 ^[^[^5^](#_ENREF_5)^]^ | 1 | 1 | 07 | 1 | | 1 | 1 | | 1 | 1 | 7 |
| Thattassery et al. 2004 ^[^[^6^](#_ENREF_6)^]^ | 1 | 1 | 0 | 1 | | 1 | 1 | | 1 | 1 | 7 |
| Emery et al. 2006 ^[^[^8^](#_ENREF_8)^]^ | 1 | 1 | 0 | 1 | | 1 | 1 | | 1 | 1 | 7 |
| Ozasa et al. 2010 ^[^[^10^](#_ENREF_10)^]^ | 1 | 1 | 0 | 1 | | 1 | 1 | | 1 | 1 | 7 |
| Siu et al. 2010 ^[^[^11^](#_ENREF_11)^]^ | 1 | 1 | 0 | 1 | | 1 | 1 | | 1 | 1 | 7 |
| Bangalore et al. 2012 ^[^[^12^](#_ENREF_12)^]^ | 1 | 1 | 0 | 1 | | 1 | 1 | | 1 | 1 | 7 |
| Bao et al. 2012 ^[^[^13^](#_ENREF_13)^]^ | 1 | 1 | 0 | 1 | | 1 | 1 | | 1 | 1 | 7 |
| Nakatani et al. 2013 ^[^[^14^](#_ENREF_14)^]^ | 1 | 1 | 0 | 1 | | 1 | 1 | | 1 | 1 | 7 |
| Bangalore et al. 2014 ^[^[^15^](#_ENREF_15)^]^ | 1 | 1 | 0 | 1 | | 1 | 1 | | 1 | 1 | 7 |
| Choo et al. 2014 ^[^[^16^](#_ENREF_16)^]^ | 1 | 1 | 0 | 1 | | 1 | 1 | | 1 | 1 | 7 |
| Yang et al. 2014 ^[^[^17^](#_ENREF_17)^]^ | 1 | 1 | 0 | 1 | | 1 | 1 | | 1 | 1 | 7 |
| Lee et al. 2015 ^[^[^20^](#_ENREF_20)^]^ | 1 | 1 | 0 | 1 | | 1 | 1 | | 1 | 1 | 7 |
| Raposeiras-Roubín et al. 2015 ^[^[^21^](#_ENREF_21)^]^ | 1 | 1 | 0 | 1 | | 1 | 1 | | 1 | 1 | 7 |
| Hioki et al. 2016 ^[^[^22^](#_ENREF_22)^]^ | 1 | 1 | 0 | 1 | | 1 | 1 | | 1 | 1 | 7 |
| Konishi et al. 2016 ^[^[^23^](#_ENREF_23)^]^ | 1 | 1 | 0 | 1 | | 1 | 1 | | 1 | 1 | 7 |
| Lee et al. 2016 ^[^[^24^](#_ENREF_24)^]^ | 1 | 1 | 0 | 1 | | 1 | 1 | | 1 | 1 | 7 |
| Dondo et al. 2016 ^[^[^26^](#_ENREF_26)^]^ | 1 | 1 | 0 | 1 | | 1 | 1 | | 1 | 1 | 7 |
| Shavadia et al. 2019 ^[^[^28^](#_ENREF_28)^]^ | 1 | 1 | 0 | 1 | | 1 | 1 | | 1 | 1 | 7 |
| Hagsund et al. 2020 ^[^[^29^](#_ENREF_29)^]^ | 1 | 1 | 0 | 1 | | 1 | 1 | | 1 | 1 | 7 |
| Cohort studies | | | | | | | | | | | |
| Rochon et al. 2000 ^[^[^4^](#_ENREF_4)^]^ | 1 | 1 | 0 | 1 | | 1 | 1 | | 1 | 0 | 6 |
| Arós et al. 2006 ^[^[^7^](#_ENREF_7)^]^ | 1 | 1 | 1 | 0 | | 1 | 0 | | 1 | 1 | 6 |
| Yamada et al. 2006 ^[^[^9^](#_ENREF_9)^]^ | 1 | 1 | 1 | 1 | | 1 | 1 | | 1 | 1 | 8 |
| Andell et al. 2015 ^[^[^18^](#_ENREF_18)^]^ | 1 | 0 | 1 | 1 | | 1 | 1 | | 1 | 1 | 7 |
| Goldberger et al. 2015 ^[^[^19^](#_ENREF_19)^]^ | 1 | 1 | 1 | 0 | | 0 | 1 | | 1 | 1 | 6 |
| Puymirat et al. 2016 ^[^[^25^](#_ENREF_25)^]^ | 1 | 1 | 1 | 1 | | 1 | 1 | | 0 | 0 | 6 |
| Hwang et al. 2019 ^[^[^27^](#_ENREF_27)^]^ | 1 | 1 | 1 | 1 | | 1 | 1 | | 0 | 1 | 7 |

a, Is the case definition OR adequate representativeness of the exposed cohort; b, representativeness of the cases OR selection of the non-exposed cohort; c, selection of controls OR ascertainment of exposure; d, definition of controls OR demonstration that outcome of interest was not present at start of study; e, comparability of cases and controls on the basis of the design or analysis OR comparability of cohorts on the basis of the design or analysis; f, ascertainment of exposure OR assessment of outcome; g, same method of ascertainment for cases and controls OR was follow-up long enough for outcomes to occur; h, non-response rate OR adequacy of follow-up of cohorts. When the selective grouping, the average or median follow-up time is less than six months, or the follow-up rate is less than 90%, corresponding to the representativeness of the exposed cohort, follow-up long enough for outcomes, or adequacy of follow-up gets 0 points. When adding up the scores, and the total mark is 9 points, 8-9 points awarded for low risk of bias, 6-7 points refer to moderate risk of bias, 1-5 points apply to high risk of bias, and 0 means a very high risk of bias.
