# Supplementary figures and images for: Long-Term Effect of β-Blocker Use on Clinical Outcomes in Postmyocardial Infarction Patients: A Systematic Review and Meta-Analysis
Source: Front Cardiovasc Med. 2022 Apr 8;9:779462. doi: 10.3389/fcvm.2022.779462 (PMC9024047; doi:10.3389/fcvm.2022.779462)

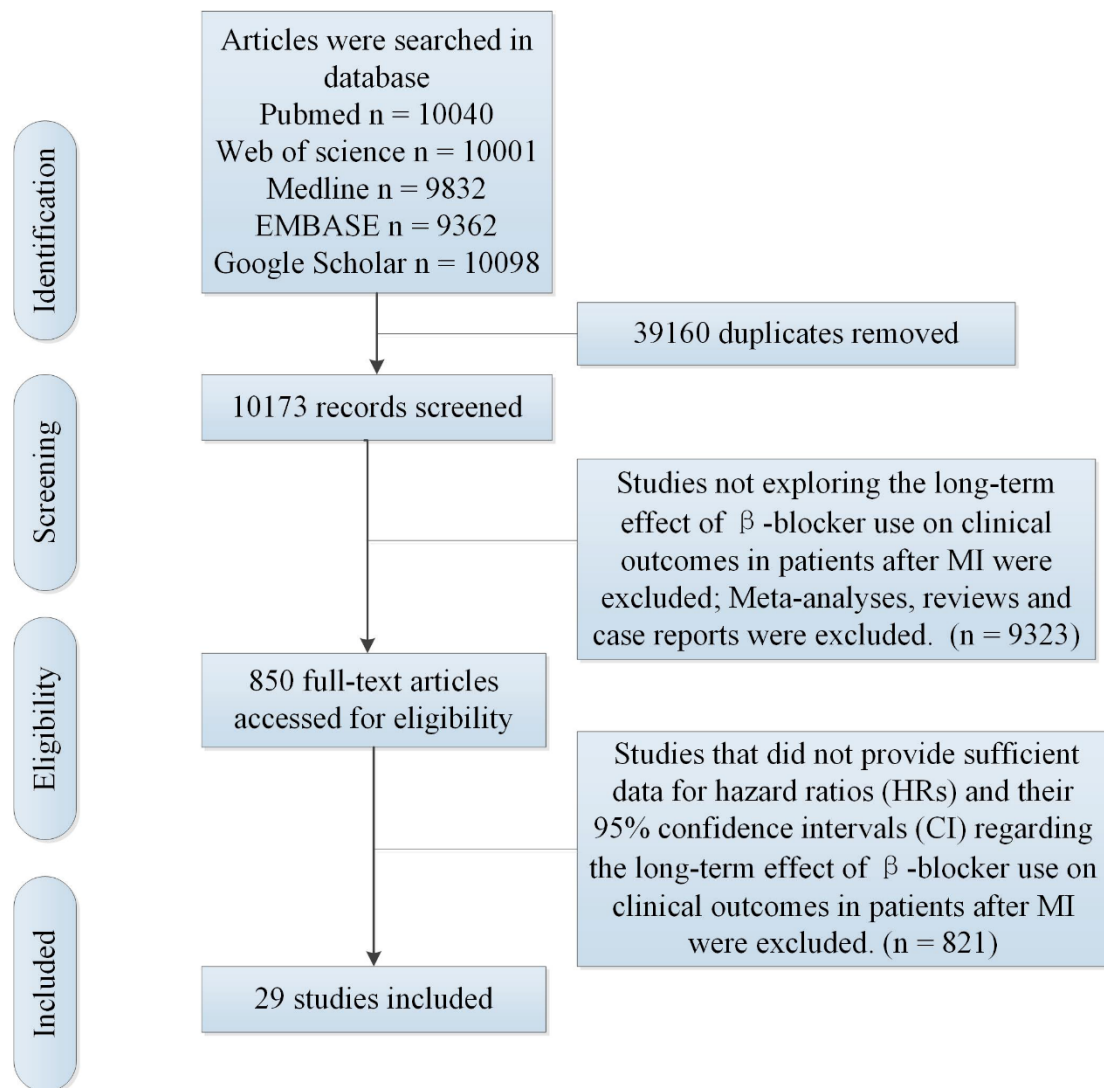

Supplementary figure 1. Flow of information through the different phases of a meta-analysis.

Supplement: Supplementary file 4 [file Image_1.pdf]
