# Supplementary material for: Long-Term Effect of β-Blocker Use on Clinical Outcomes in Postmyocardial Infarction Patients: A Systematic Review and Meta-Analysis
Source: Front Cardiovasc Med. 2022 Apr 8;9:779462. doi: 10.3389/fcvm.2022.779462 (PMC9024047; doi:10.3389/fcvm.2022.779462)
Supplement: Supplementary file 5 [file Image_2.pdf]

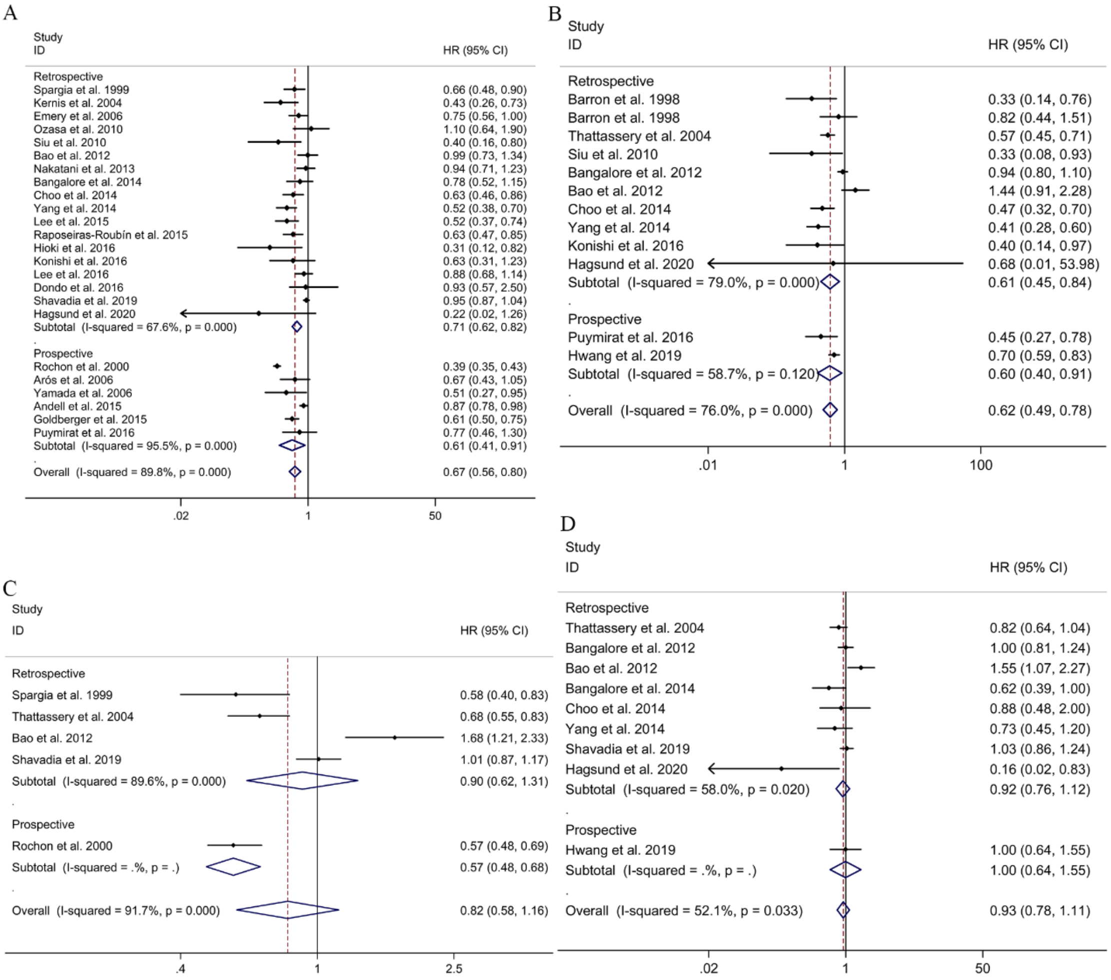

Supplementary figure 2. Subgroup studies exploring long-term effect of β-blocker use on all-cause mortality (A), cardiovascular mortality (B), risk of hospitalization for HF (C) and risk of recurrent MI (D) in patients after MI in different types of studies. Abbreviations: CI, confidence interval; HF, heart failure; HR, hazard ratio; MI, myocardial infarction.
