# Supplementary material for: Long-Term Effect of β-Blocker Use on Clinical Outcomes in Postmyocardial Infarction Patients: A Systematic Review and Meta-Analysis
Source: Front Cardiovasc Med. 2022 Apr 8;9:779462. doi: 10.3389/fcvm.2022.779462 (PMC9024047; doi:10.3389/fcvm.2022.779462)
Supplement: Supplementary file 6 [file Image_3.pdf]

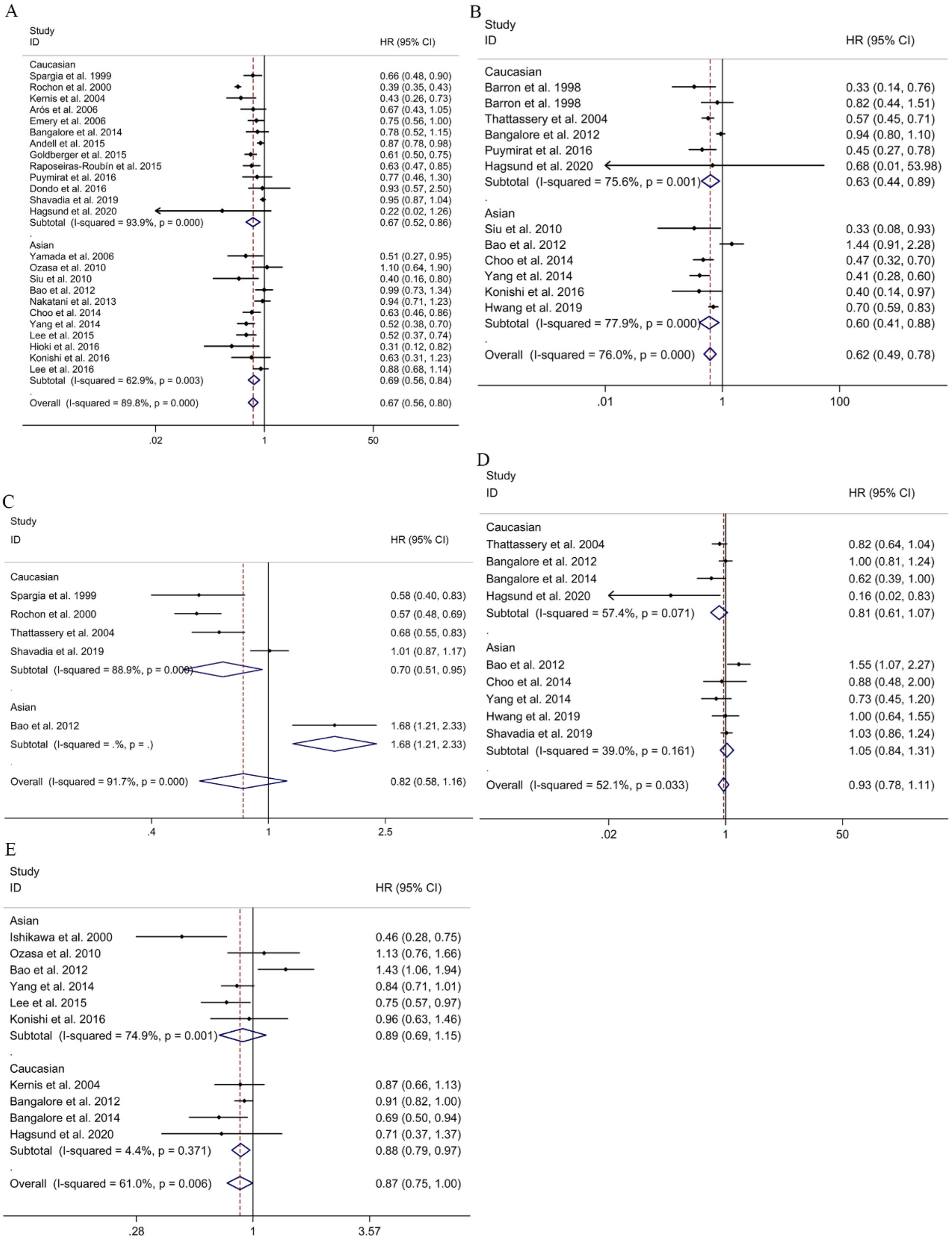

Supplementary figure 3. Subgroup studies exploring long-term effect of β-blocker use on all-cause mortality (A), cardiovascular mortality (B), risk of hospitalization for HF (C), risk of recurrent MI (D) and risk of MACE (E) in patients after MI in Caucasian and Asian groups. Abbreviations: CI, confidence interval; HF, heart failure; HR, hazard ratio; MACE, major adverse cardiac events; MI, myocardial infarction.
