# Supplementary material for: Long-Term Effect of β-Blocker Use on Clinical Outcomes in Postmyocardial Infarction Patients: A Systematic Review and Meta-Analysis
Source: Front Cardiovasc Med. 2022 Apr 8;9:779462. doi: 10.3389/fcvm.2022.779462 (PMC9024047; doi:10.3389/fcvm.2022.779462)
Supplement: Supplementary file 7 [file Image_4.pdf]

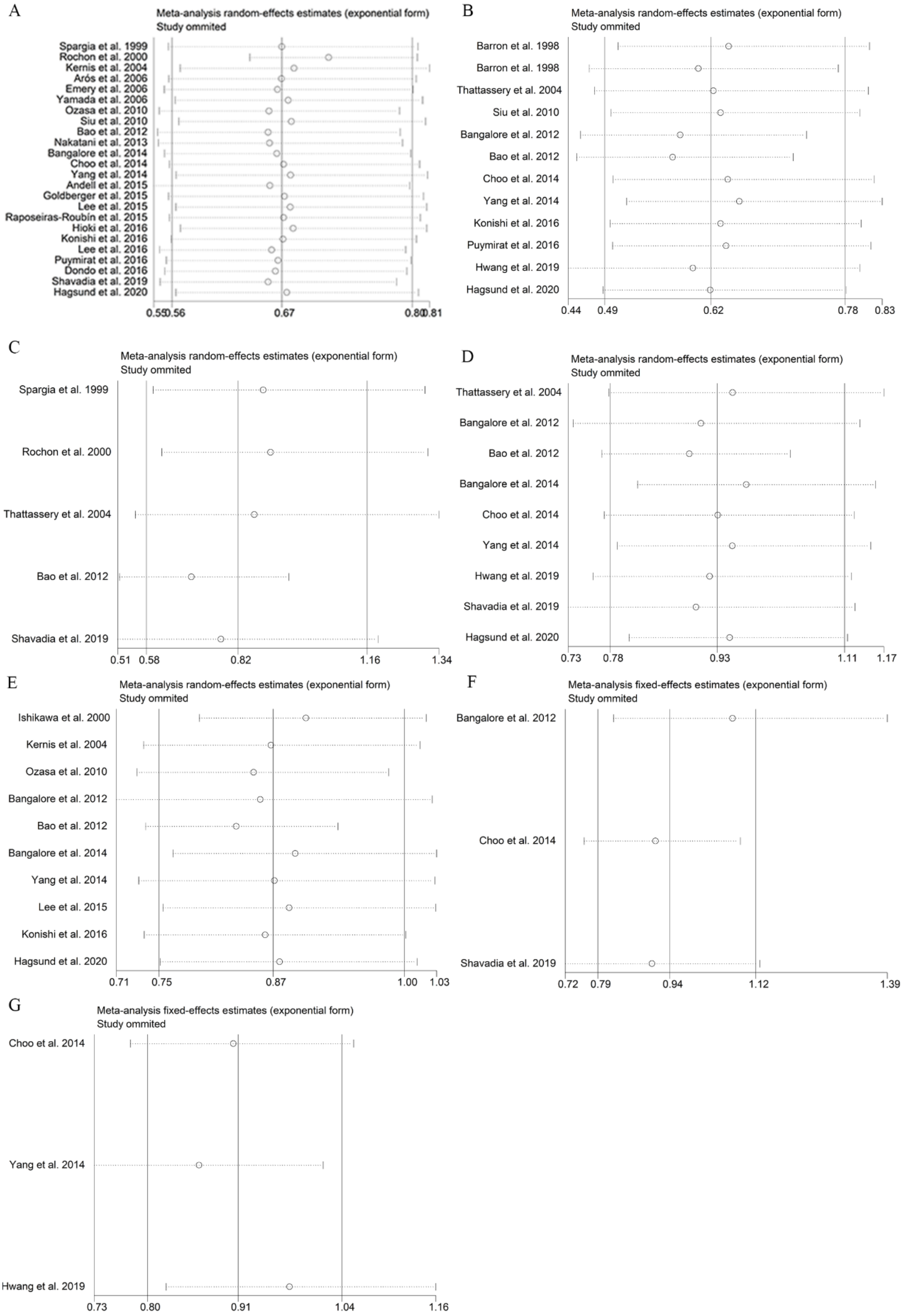

Supplementary figure 4. Sensitivity analyses exploring long-term effect of β-blocker use on all-cause mortality (A), cardiovascular mortality (B), risk of hospitalization for HF (C), risk of recurrent MI (D), risk of MACE (E), risk of stroke (F) and risk of repeat revascularization (G) in patients after MI. Abbreviations: CI, confidence interval; HF,

heart failure; HR, hazard ratio; MACE, major adverse cardiac events; MI, myocardial infarction.
