# Supplementary material for: Long-Term Effect of β-Blocker Use on Clinical Outcomes in Postmyocardial Infarction Patients: A Systematic Review and Meta-Analysis
Source: Front Cardiovasc Med. 2022 Apr 8;9:779462. doi: 10.3389/fcvm.2022.779462 (PMC9024047; doi:10.3389/fcvm.2022.779462)
Supplement: Supplementary file 10 [file Image_7.pdf]

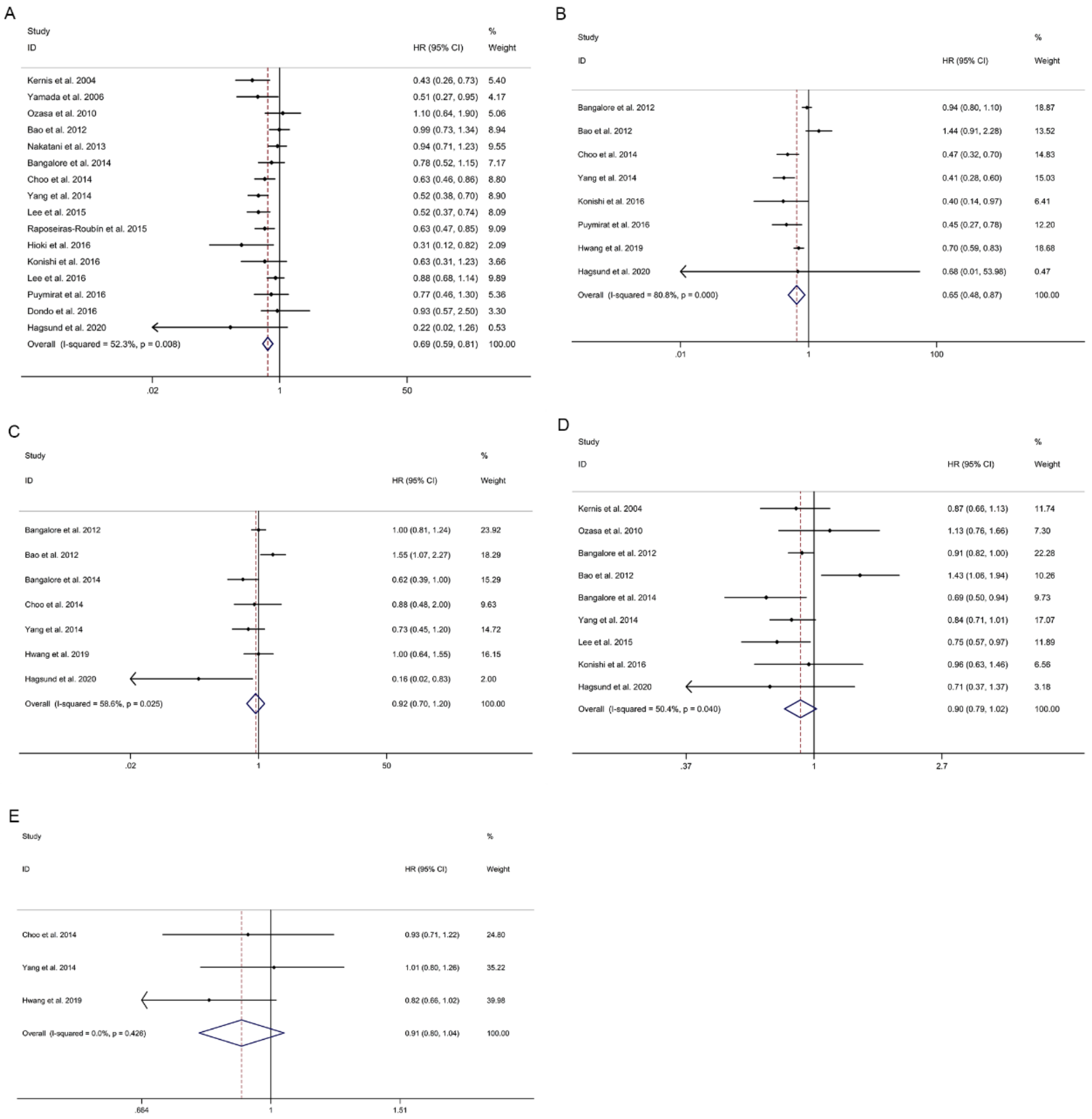

Supplementary figure 7. Forest plots exploring long-term effect of β-blocker use on all-cause mortality (A), all-cause mortality (B), risk of recurrent MI (C), risk of MACE (D), risk of repeat revascularization (E) in patients after MI with low EF. Abbreviations: CI, confidence interval; HR, hazard ratio; MACE, major adverse cardiac events; MI, myocardial infarction.
